# Comparison of point-of-care peripheral perfusion assessment using pulse oximetry sensor with manual capillary refill time: clinical pilot study in the emergency department

**DOI:** 10.1186/s40560-019-0406-0

**Published:** 2019-11-27

**Authors:** Koichiro Shinozaki, Lee S. Jacobson, Kota Saeki, Hideaki Hirahara, Naoki Kobayashi, Steve Weisner, Julianne M. Falotico, Timmy Li, Junhwan Kim, Lance B. Becker

**Affiliations:** 1 The Feinstein Institute for Medical Research, Northwell Health, 350 Community Dr., Manhasset, NY 11030 USA; 2 Department of Emergency Medicine, North Shore University Hospital, Northwell Health, Manhasset, NY USA; 3 Nihon Kohden Innovation Center, Cambridge, MA USA; 4 Nihon Kohden Corporation, Tokyo, Japan

**Keywords:** Capillary refill time, Visual assessment, Peripheral perfusion status, Outcome prediction

## Abstract

**Background:**

Traditional capillary refill time (CRT) is a manual measurement that is commonly used by clinicians to identify deterioration in peripheral perfusion status. Our study compared a novel method of measuring peripheral perfusion using an investigational device with standardized visual CRT and tested the clinical usefulness of this investigational device, using an existing pulse oximetry sensor, in an emergency department (ED) setting.

**Material and methods:**

An ED attending physician quantitatively measured CRT using a chronometer (standardized visual CRT). The pulse oximetry sensor was attached to the same hand. Values obtained using the device are referred to as blood refill time (BRT). These techniques were compared in its numbers with the Bland-Altman plot and the predictability of patients’ admissions.

**Results:**

Thirty ED patients were recruited. Mean CRT of ED patients was 1.9 ± 0.8 s, and there was a strong correlation with BRT (*r* = 0.723, *p* < 0.001). The Bland-Altman plot showed a proportional bias pattern. The ED physician identified 3 patients with abnormal CRT (> 3 s). Area under the receiver operator characteristic curve (AUC) of BRT to predict whether or not CRT was greater than 3 s was 0.82 (95% CI, 0.58–1.00). Intra-rater reliability of BRT was 0.88 (95% CI, 0.79–0.94) and that of CRT was 0.92 (0.85–0.96). Twelve patients were admitted to the hospital. AUC to predict patients’ admissions was 0.67 (95% CI, 0.46–0.87) by BRT and 0.76 (0.58–0.94) by CRT.

**Conclusions:**

BRT by a pulse oximetry sensor was an objective measurement as useful as the standardized CRT measured by the trained examiner with a chronometer at the bedside.

## Introduction

Capillary refill time (CRT) is a simple and non-invasive test typically used to assess peripheral perfusion status at the bedside. A prolonged CRT suggests a decrease in peripheral perfusion and is used to identify hemodynamically compromised patients in critical care [1–3]. However, CRT is a relatively subjective test given that clinicians rely on visual assessments to perform the measurement. Since the reliability of traditional CRT tests has been questioned over the last few decades [4–6], there is a great demand for the creation of objective methods to assess peripheral blood perfusion [5, 7].

Pulse oximetry utilizes spectroscopic technology to noninvasively measure oxygen saturation by measuring changes in light absorption of oxy/deoxy hemoglobin [8, 9], and there is a potential use of this technology as point-of-care testing in multiple clinical situations. We developed an investigational device that attaches a normal pulse oximetry sensor to the fingertip with the goal of providing clinicians with the means to collect alternative measures to traditional CRT tests. Our device calculates the time it takes for blood to return to the fingertip after it is released from compression [10, 11] by algorithmically analyzing the light intensity waveform of the pulse oximetry sensor transmitted through the fingertip. Since the mechanisms to assess peripheral blood perfusion are different from traditional CRT tests, we differentiate the measurement of our device from CRT and name it blood refill time (BRT) in this report.

Previous studies investigated other objective measurements of peripheral perfusion status in critical care settings. For example, Bakker and Lima’s group examined the reliability of standardized manual CRT in conjunction with the objective measurement forearm-to-fingertip skin-temperature gradient (Tskin-diff) [2]. In their clinical study, including 111 postoperative patients, they reported that the predictability of postoperative complications was higher by subjective CRT than with objective Tskin-diff [3]. Near-infrared spectroscopy (NIRS) can also be used to assess peripheral perfusion status. Lima [12] reported that peripheral vasoconstriction induced by body surface cooling altered tissue oxygen saturation as measured by NIRS attached to the thenar eminence. However, there have not yet been any studies evaluating BRT in the emergency department (ED).

The purpose of our study was to compare BRT and CRT in an ED setting. There are two methodological differences in manual CRT: strict/advanced procedure or normal/classic procedure. The strict procedure is a quantitative measurement to standardize CRT, while the classic procedure is qualitative. The strict procedure is performed by a well-trained examiner with a chronometer. This is considered a separate CRT measurement from the originally introduced classic CRT and the standardized CRT has shown high reliability in the previous clinical studies [1–3]. Therefore, the strict procedure was used for measuring standardized CRT in our study. One of the important applications of objective peripheral perfusion assessments is a triage tool in the ED. Early recognition of patients who need admission is imperative to optimize resources in ED settings. Therefore, we also compare BRT and CRT in their predictability of patients’ admissions.

## Methods

### Study design and patients

This was a cross-sectional study conducted in the ED of a suburban, quaternary care teaching hospital. ED patients who met the following inclusion criteria were recruited: aged 18 years or older and able to provide written informed consent. Exclusion criteria were the following: pregnancy, current imprisonment, cognitive impairment, a determination of instability by the clinical team, and finger, hand, or forearm anatomical anomalies or diseases that interfered with attaching a pulse oximetry sensor.

The study protocol was approved by the Institutional Review Board of Northwell Health (study no. 17-0805). Informed consent for participation was obtained from all patients prior to the completion of any study procedures.

### Measurements

**Fig. 1:**
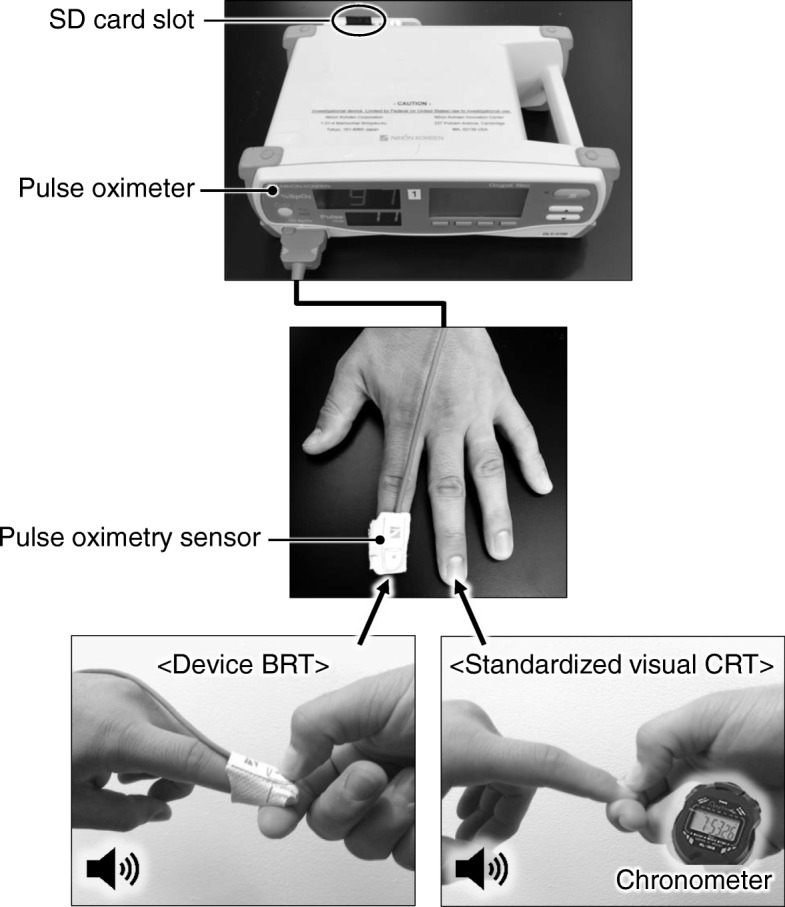
Schema of the device BRT and the standardized visual CRT measurements. CRT was measured using a chronometer. The examiner compressed the fingertip for 5 s, signaled by “start compression” and “release compression” beep sounds. When the fingertip was released from compression, the examiner began the standardized visual CRT measurement. A pulse oximetry sensor was applied, and the fingertip was compressed and released 5 s after starting compression. There is a SD card slot on the back panel of the device. The waveforms of the light intensity were stored in the SD card. The data was calculated by a pre-fixed algorithm

An ED attending physician quantified the patient’s CRT using a chronometer. In this study, only one ED attending physician manually measured quantified CRT from all patients. The ED attending physician was not involved in the patients’ clinical care. The subject’s most accessible hand and fingers were used. The physician compressed the fingertip of the subject’s second or third finger for 5 s, signaled by “start compression” and “release compression” beep sounds. When the fingertip was released from compression, the physician began the standardized visual CRT measurement (Fig. 1). The physician held the chronometer in the hand that did not perform the compression and used this chronometer to measure CRT (Method 1).

The investigational device is a non-invasive, peripheral hemodynamic monitor composed of two components: a pulse oximeter (modified Model OLV-3100, Nihon Kohden Corporation, Tokyo, Japan) and pulse oximetry sensor (TL-271 T/TL-271 T3, Nihon Kohden Corporation, Tokyo, Japan). We used one wavelength (infrared light: 940 nm) to trace the change of blood volume at the fingertip. The light intensity transmitted through the fingertip increases during compression as hemoglobin in the blood, which is the major absorber of the light, is squeezed out of the fingertip. There is a SD card slot on the back panel of the device (Fig. 1). There is no output source, and thus, real-time BRT calculation is not available on the current model of the device. The waveforms of the light intensity were recorded by the device every 16 ms, and the data was stored in the SD card. The ordinary Model OLV-3100 does not have the function to store the light intensity data into the SD card, and so the software and hardware modifications were added for the study purpose.

A pulse oximetry sensor was applied to the same hand of the subject used in Method 1 and was attached to either the second or third fingertip, depending upon which fingertip was compressed in Method 1 (Fig. 1). For example, if the second fingertip was used in Method 1, then the sensor was applied to the third fingertip. With the sensor on it, the physician compressed the fingertip for 5 s signaled by beep sound from the device, and the device recorded the waveforms of the light intensity corresponding to the compression and the release (Method 2). Methods 1 and 2 were performed alternately and were repeated three times each for a total of six compressions per subject. The attending physician was blinded from BRT values.

The data was calculated by a pre-fixed algorithm and the investigator, who calculated the data, was blinded from the results. The compression phase is followed by the release phase during which light intensity returns to its original level. The curve displaying the recovery phase of the intensity waveform (intensity returning to its original level) is modeled as an exponential decay using the least squares method. The time to achieve a 90% return of intensity was reported as BRT. These methods are more extensively described elsewhere [10, 11]. Data on the patient’s age, gender, race, Fitzpatrick skin tone scale, body weight, height, past medical history (diabetes mellitus, hypertension, smoking, medications, heart disease, lung disease, others), body temperature, hemoglobin (Hb), hematocrit (Ht), blood lactate levels (if available), complete blood count (CBC), and lab data (C-reactive protein, CRP; erythrocyte sedimentation rate, ESR; aspartate aminotransferase, AST; alanine aminotransferase, ALT; lactate dehydrogenase, LDH; creatinine, Cre; blood urea nitrogen, BUN; total protein, TP; albumin, Alb, if available) were collected from the medical chart. Tskin-diff was calculated as the difference between fingertip temperature and forearm temperature. A non-contact infrared thermometer (NUB8380H Non-contact Infrared Thermometer, Nubee, CA, USA) measured the surface temperature of the patient’s fingertip and forearm on the radial side, midway between the elbow and the wrist. Tc-diff was calculated as the difference between fingertip temperature and body temperature. Room temperature was measured during each patient enrollment.

## Statistical analysis

The sample size of 30 was determined following the general flat rule cited by Browne [13, 14], since this was the preliminary pilot study for a further clinical trial. The mean and standard deviation or median and interquartile range were reported appropriately. The mean of the three repeated BRT/CRT measurements was considered the value for each patient. Pearson’s correlation coefficient was calculated to assess the correlation between each method and patient information. Intra-rater reliability was assessed using intra-class correlation coefficients. The Bland-Altman plot was used to compare BRT and CRT. The differences between the two techniques were plotted against the averages of the two techniques. Receiver operator characteristic (ROC) curve analysis was performed, and area under the curve (AUC) was analyzed to demonstrate the predictability of the measurements. A 3-s cutoff value was used for standardized visual CRT measurements as it was referred from the previous study [1]. An optimal cutoff value for BRT to predict patient admission to the hospital was calculated by assessing the area under the ROC curve. A two-tailed *p* value of < 0.05 was considered statistically significant. All calculations were performed with SPSS Statistics ver 22 for Mac (IBM Corp., Armonk, NY).

## Results

Thirty adult ED patients voluntarily participated, and their demographic data are displayed in Table 1. The admission rate of study patients to the hospital was 40%. Mean fingertip skin temperature was 27.8 ± 3.0 °C. Room temperature was 23.4 ± 1.0 °C. The ED physician quantitatively determined that 3 patients had CRT values greater than 3.0 s. All 3 of these patients were admitted to the hospital.

**Table 1:** Demographic data of the patients

	*n* = 30
Age, years	58.6 ± 19.8
Gender, male (%)	13 (43)
Race, *n* (%)	
White	17 (57)
Black or African American	10 (33)
Asian	2 (7)
Other/multiracial	1 (3)
Past medical history, *n* (%)	
Diabetes mellitus	6 (20)
Hypertension	13 (43)
Smoking	6 (20)
Heart disease	9 (30)
Lung disease	1 (3)
Patient type	
Medical, *n* (%)	24 (80)
Surgical, *n* (%)	6 (20)
Infection, *n* (%)	11 (37)
Temperature, °C	
Fingertip temperature	27.8 ± 3.0
Forearm temperature	32.5 ± 1.0
Body temperature	37.0 ± 0.5
Initial vital signs	200
Heart rate, BPM	92 ± 17
Respiratory rate, BPM	18 ± 2
Systolic blood pressure, mmHg	131 ± 24
Diastolic blood pressure, mmHg	77 ± 13
Oxygen saturation, %	98 (97, 99)
Shock status	
Systolic blood pressure < 90 mmHg, *n* (%)	0 (0)
Lactate > 2.0 mmol/L, *n* (%)	4 (13)
Interventions	
Vasopressor use, *n* (%)	0 (0)
Inotropic support, *n* (%)	0 (0)
Bolus fluid administered, *n* (%)	22 (73)
Volume of fluid, mL	1750 (1000, 2938)
Bolus before cap refill, *n* (%)	18 (82)
Bolus after cap refill, *n* (%)	4 (18)
Admission to the hospital, *n* (%)	12 (40)

Mean and standard deviation, median and interquartile, or number and proportion are shown

### Standardized visual CRT and device BRT

**Fig. 2:**
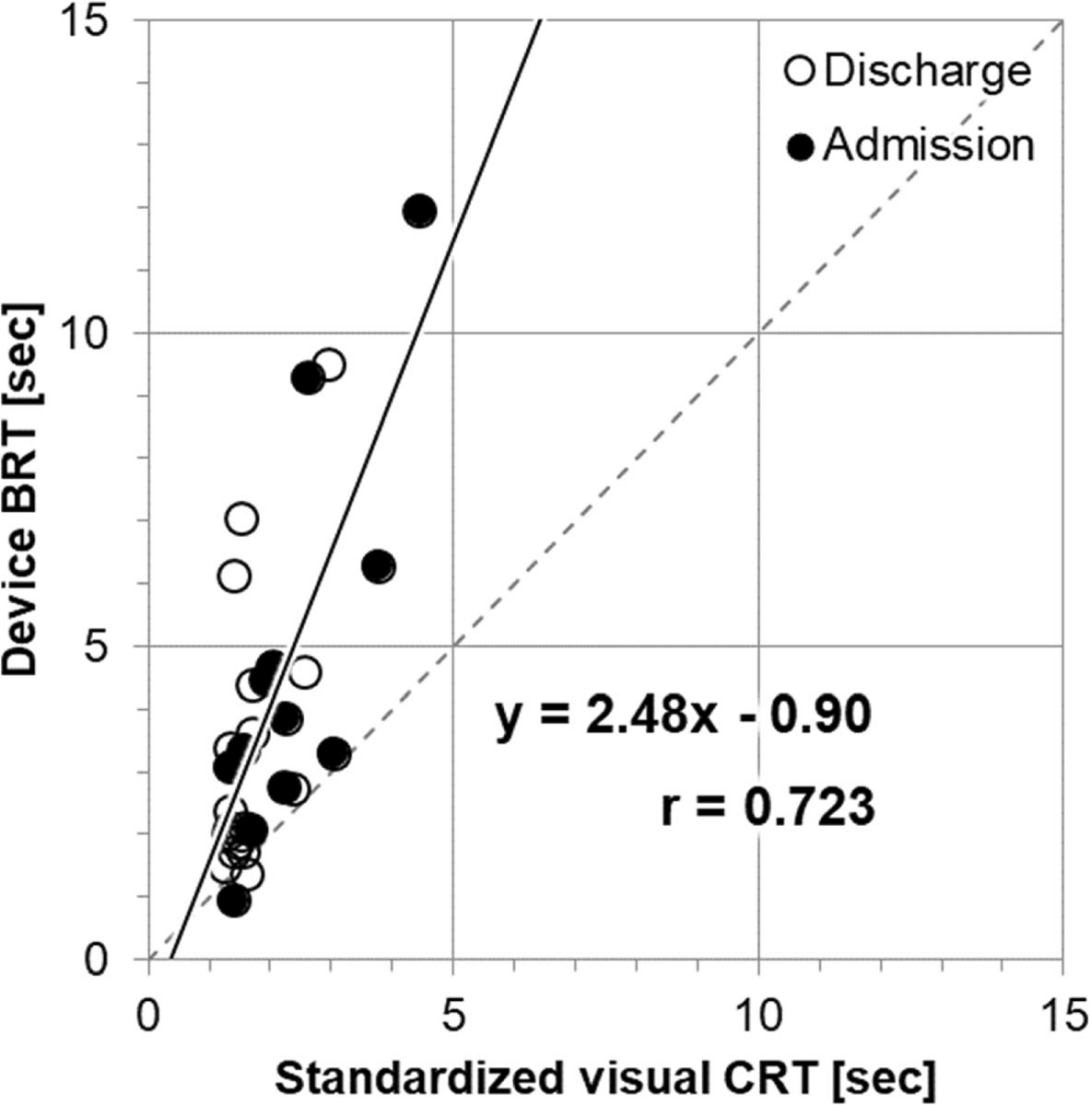
Scatter plot of device BRT as a function of standardized visual CRT. There was a strong correlation between CRT and BRT (Pearson correlation coefficient: 0.72, *p* <0.001). Black dots represent patients who were required admission, and white circular dots are patients who were discharged. BRT, blood refill time; CRT, capillary refill time

**Fig. 3:**
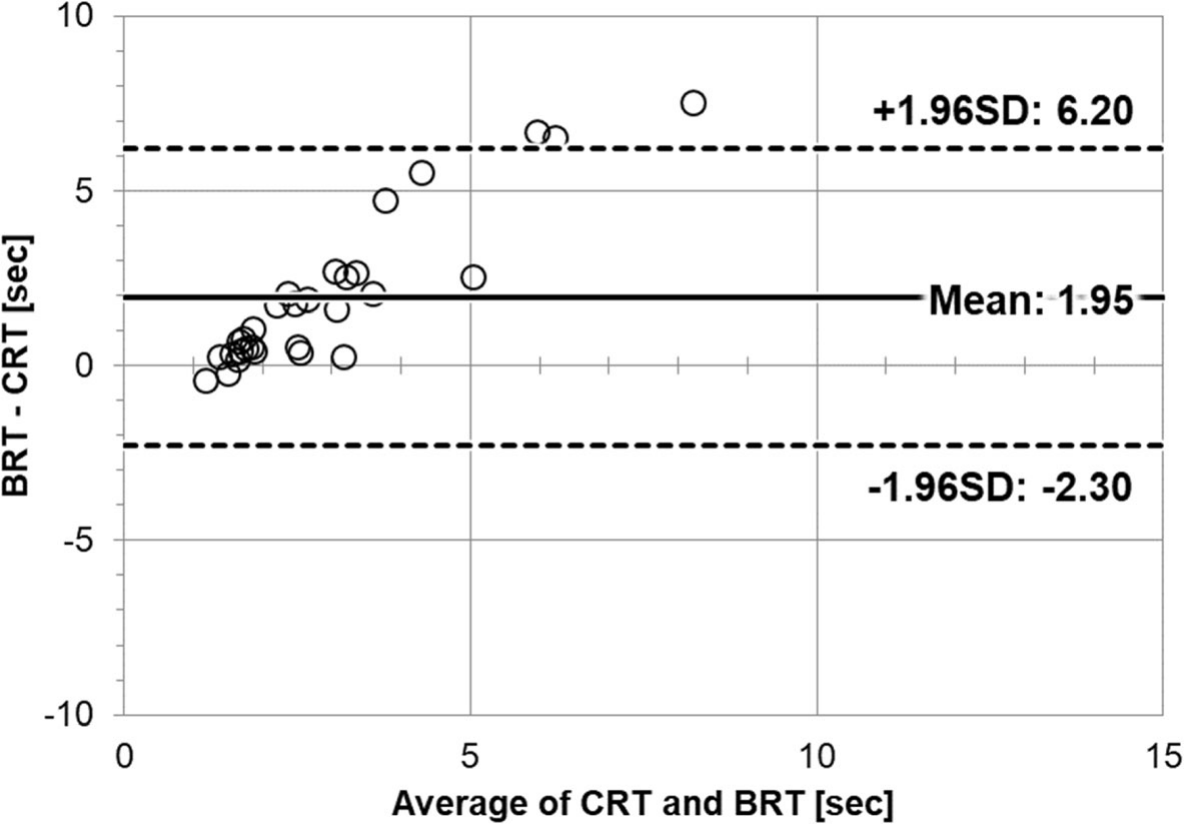
The Bland-Altman Plot. The differences between the two techniques were plotted against the averages of the two techniques since there were no gold standard techniques. A proportional bias pattern was found between BRT and CRT

Standardized visual CRT by the attending physician (Method 1) ranged from 1.24 to 4.44 s with a mean of 1.93 ± 0.78 s. Device BRT (Method 2) ranged from 0.96 to 11.97 s with a mean of 3.88 ± 2.66 s. There was a strong correlation between CRT and BRT (Pearson correlation coefficient: 0.72, *p* < 0.001). Figure 2 depicts the scatter plot of BRT as a function of CRT. BRT showed longer refill times than standardized visual CRT. Figure 3 is the Bland-Altman plot of the two methods. The differences between the two techniques were plotted against the averages of the two techniques since there was no gold standard technique. A proportional bias pattern was found between BRT and CRT.

Intra-observer reliability, measured by an intra-class coefficient of 3-time CRT measurements, was 0.79 (95% CI, 0.66–0.88) for single measure and 0.92 (0.85–0.96) for average measures. That of BRT was 0.72 (0.55–0.84) and 0.88 (0.79–0.94), respectively.

**Fig. 4:**
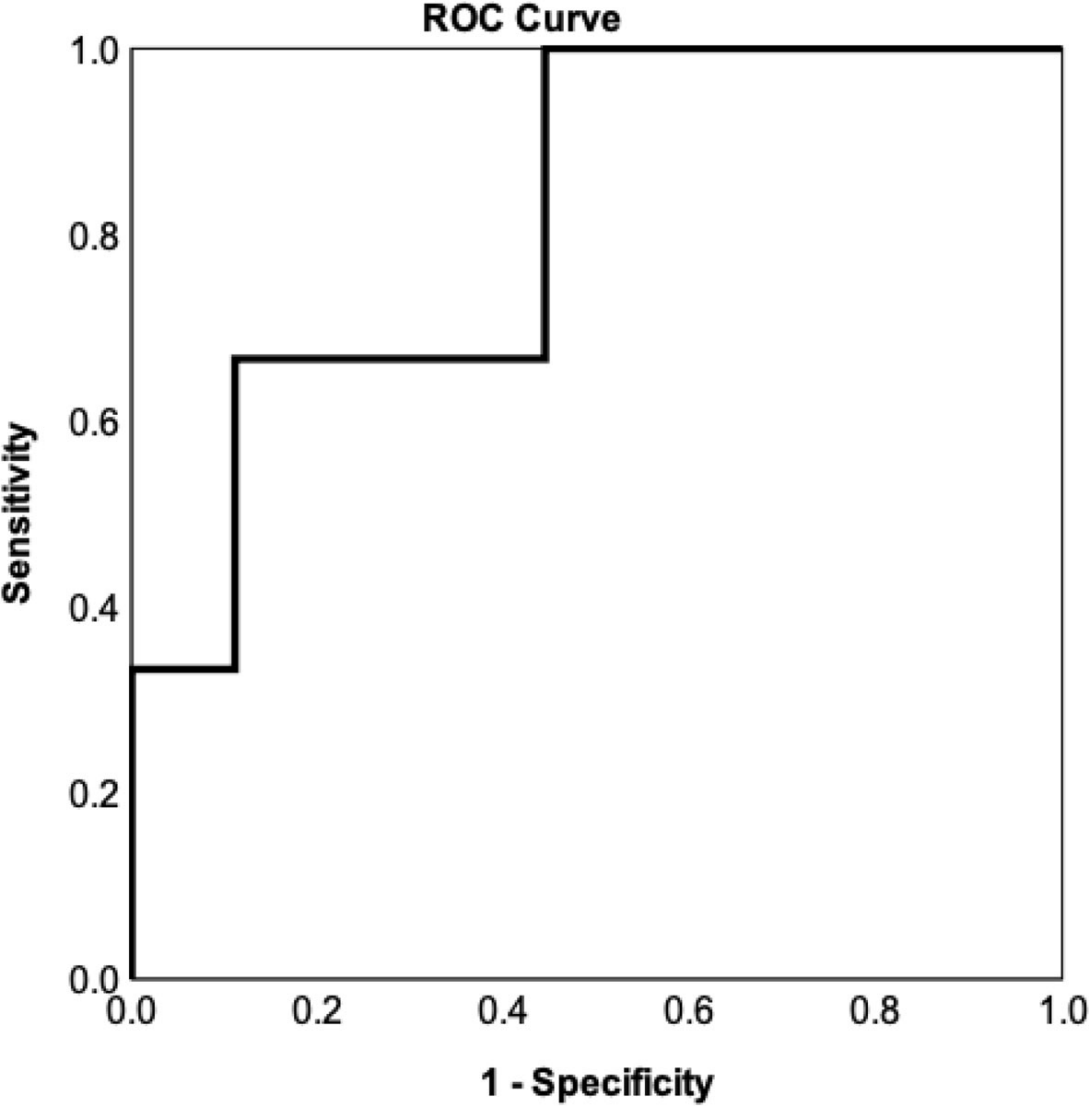
Receiver operating curve of device BRT to predict abnormal standardized visual CRT. ROC analysis of BRT was performed to predict whether or not standardized visual CRT by the attending physician was greater than or less than 3.0 s. The area under the ROC curve was 0.82 (95% CI, 0.58–1.00). ROC, receiver operating curve; BRT, blood refill time; CRT, capillary refill time

The ED physician quantitatively determined that 3 patients had abnormal CRT values. ROC analysis of BRT was performed to predict whether or not standardized visual CRT by the attending physician was greater than or less than 3.0 s. The area under the ROC curve was 0.82 (95% CI, 0.58–1.00) and is shown in Fig. 4.

### Correlation of standardized visual CRT or device BRT with other data

Standardized visual CRT showed positive correlations with patient’s age (*r* = 0.45, *p* = 0.012), BUN (*r* = 0.42, *p* = 0.031), and Cre (*r* = 0.60, *p* = 0.001) and showed negative correlations with Hb (*r* = − 0.59, *p* = 0.002), Ht (*r* = − 0.50, *p* = 0.011), RBC (*r* = − 0.48, *p* = 0.016), and Alb (*r* = − 0.58, *p* = 0.002). There was no correlation with Tskin-diff (*r* = 0.09, *p* = 0.653) or Tc-diff (*r* = 0.07, *p* = 0.714). BRT showed positive correlations with patient’s age (*r* = 0.58, *p* = 0.001), BUN (*r* = 0.41, *p* = 0.038), and Cre (*r* = 0.66, *p* < 0.001) and showed negative correlations with fingertip temperature (*r* = − 0.38, *p* = 0.040), RBC (*r* = − 0.43, *p* = 0.032), Alb (*r* = − 0.40, *p* = 0.043). There was a trend of correlation but no statistical significance with Tskin-diff (*r* = 0.34, *p* = 0.068) and Tc-diff (*r* = 0.35, *p* = 0.069).

### Prognostic value of standardized visual CRT and device BRT on patient admission

Twelve patients were admitted. Standardized visual CRT of admitted patients was 2.35 ± 0.97 and percent coefficient of variation (%CV) was 41%. Standardized visual CRT of discharged patients was 1.65 ± 0.47 and %CV was 29%. BRT of admitted patients was 4.69 ± 3.14 and %CV was 67%. BRT of discharged patients was 3.35 ± 2.23 and %CV was 67%.

**Fig. 5:**
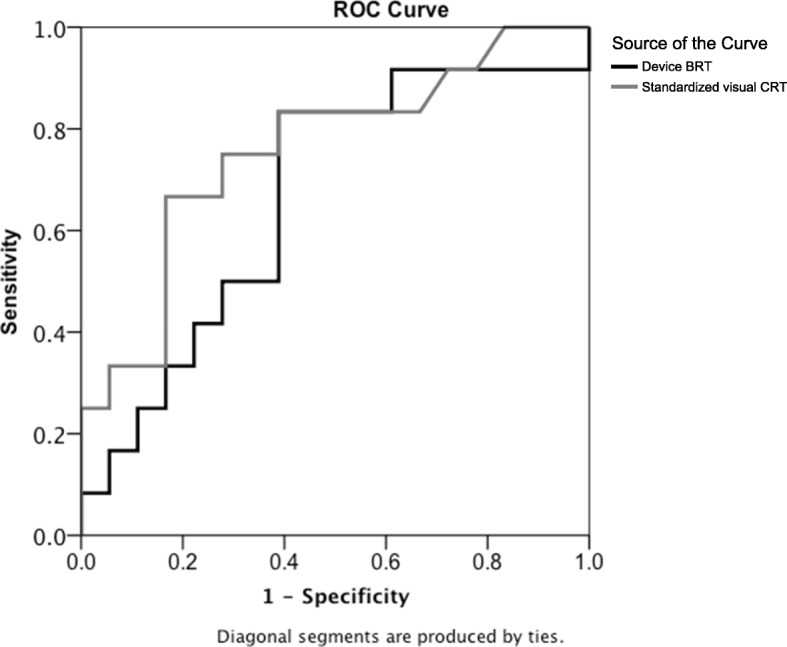
Receiver operating curve of device BRT and standardized visual CRT to predict ED patients’ admissions. The area under the ROC curve of standardized visual CRT was 0.76 (95% CI, 0.58–0.94) and that of device BRT was 0.67 (95% CI, 0.46–87). ROC, receiver operating curve; ED, emergency department; BRT, blood refill time; CRT, capillary refill time

ROC curve analysis to predict patient admission was performed with standardized visual CRT and device BRT. AUC of standardized visual CRT was 0.76 (95% CI, 0.58–0.94) and that of device BRT was 0.67 (95% CI, 0.46–0.87). The ROC curves are seen in Fig. 5.

The optimal cutoff value (left upper corner) of BRT to predict patient admission was 2.74 s. There were 17 patients who had BRT over 2.74 s, and 10 out of 17 patients were admitted. There were 13 patients whose BRT was 2.74 s or lower, and 11 out of 13 patients were discharged. Sensitivity, specificity, and positive and negative predictive values using this cutoff value were 83.3%, 61.1%, 58.8%, and 84.6%, respectively.

## Discussion

A convenience sample of 30 adult ED patients was enrolled at a suburban, quaternary care US teaching hospital. We evaluated multiple values, including predictability for patients’ admissions, and compare between the newly developed point-of-care testing, BRT, and the manual CRT measurement. There were strong correlations between BRT and CRT, and there were no differences in predictability between these techniques. The results of this study thus provide empirical evidence to answer the question: does objective BRT become an alternative to the manual CRT? Interestingly, indicators which were found to be correlated with BRT are factors that can also be associated with the patient’s dehydration condition, e.g., decreased RBC and Alb and increased Cre and BUN.

Alsma [6] studied visually assessed CRT at the fingertip following 5-s compressions and found that the intra-class coefficient (ICC) of quantified CRT was 0.52 (95% CI, 0.49–0.56) and the kappa value of qualified CRT was 0.40 (95% CI, 0.36–0.45), and stated that inter-observer reliability is, at best, moderate in the ED/hospital. Gorelick [4] reported fair inter-observer reliability with an ICC of 0.70 (95% CI, 0.56–0.85) and kappa of 0.54 (95% CI, 0.33–0.73) in the pediatric ED. However, in contrast to other reports, van Genderen [3] reported excellent to good inter-observer reliability between two raters showing a kappa value of 0.91 (95% CI, 0.80–0.97) and 0.74 (95% CI, 0.52–0.89) from different postoperative days. Importantly, Pickard [5] highlighted the possibility that the strict methods utilized to assess CRT, such as timing refill with a chronometer, may limit some reports from accurately representing usual clinical practice. Our study procedure of the manual CRT was the strict/quantified measure that was the same as van Genderen’s study. Manual CRT measurement is subjective; therefore, its reliability is limited and this measure may not be appropriately used as a gold standard in clinical settings. However, a careful measurement of CRT by trained examiners with a chronometer increases the reliability. We validated the reliability of standardized visual CRT by using image analysis software and, in fact, a strong correlation between these measures was found in our preliminary (data not shown). This strict method has been acknowledged as a robust study tool for the evaluation of peripheral perfusion status [2, 3].

More importantly, the strict CRT measurement recently demonstrated a strong evidence of peripheral perfusion guided treatment in sepsis [1, 15]. In our study, one trained examiner performed every single measurement and the time for finger compression was strictly controlled by a beep sound. A chronometer was used, which was the key to measuring reliable CRT. In addition, we recently reported that the training level of examiners positively impacts the accuracy and reliability of human visual assessment that is essential for measuring reliable CRT [16]. Therefore, in our study setting, the manual CRT was safely used as a study tool being compared with our novel BRT measurement.

van Genderen [3] reported the best prognostic value by standardized visual CRT assessment followed by slightly inferior prognostic value by other objective assessments, such as Tskin-diff. We also found a trend of correlation between BRT and Tskin-diff, while there was almost no correlation between CRT and Tskin-diff. Both BRT and Tskin-diff are objective measurements, but CRT is not. We did not compare BRT with lactate levels due to small numbers of patients with elevated lactate. Although standardized visual CRT assessment is a good prognostic indicator in critical care [2], the subjective nature of the assessment may lead to variability in performance between individual clinicians. Therefore, the development of clinical devices that allows for objective assessments is necessary in order to study the prognostic value of peripheral perfusion status on patient outcomes.

In this clinical study, we found a weak negative correlation between BRT values and low fingertip temperature. It is important to consider that clinical patients might have several confounders. Future studies would thus be of use in order to identify clinical factors that are associated with prolonged BRT, such as conditions that alter peripheral vascular auto regulation (sympathetic nerve activity, stress factors, pain, cold temperature, etc.). Since device BRT measurements are more objective than standardized visual CRT assessments, there is potential for increased performance by the device if the confounders that affect the device measurements are taken into consideration.

Clinically, there are two potential ways to use BRT: as a triage tool in the ED and/or as a screening tool for the severity of patients in critical care. In the ED, it is of paramount importance for clinicians to quickly disposition patients; therefore, an accurate triage is essential to optimize resources in the ED. The predictability of admissions with BRT and/or with other objective measurements needs to be evaluated in a future multi-center trial. Our data from this pilot study may be used to calculate the sample size needed for the future trial. Our present work did not include patients, whose outcome was death or whose hemodynamic status was in shock. A future study may wish to be conducted to evaluate BRT to assess the severity of patients. The number of patients needed should be large enough, which depends on the mortality or the shock rate of the target population. The sample size will be based on the rates of the target outcomes plus the difference of BRT between the groups, such as survival vs. death or shock vs. non-shock. Having data of percent coefficient of variation (standard deviation divided by mean number) is a key element to calculate the sample size for future analysis. There was a trend of proportional bias found in the Bland-Altman plot. The sicker patients may show proportionally increased number of BRT. However, percent coefficient of variation does not change if the trend of proportional bias remains in the sicker patients. There have been few studies using BRT in patients; therefore, the data provided from our study will be valuable for researchers wishing to conduct studies with BRT in the future.

This study has several limitations that should be acknowledged. Firstly, the study sample is limited. In order to identify independent prognostic factors, a validation study with greater sample numbers, multiple regression analysis, and adjustment for multiple confounders are necessary. Secondly, we used a convenience sample of ED patients, which may not be truly representative of the general ED population. However, some outcomes measured in our report, such as sensitivity and specificity, do not vary according to disease prevalence and therefore are not altered by the patient population from which our convenience sample is comprised. Thirdly, patients who required admission might not have peripheral perfusion failure. In order to evaluate the clinical value of BRT, more important patient outcomes, such as mortality or shock status, may wish to be tested in a future trial. However, this study was not conducted to evaluate the diagnostic accuracy of BRT to identify hemodynamically compromise patients. This is a significant study limitation but does not affect the conclusion of our present work. Fourthly, we chose 5 s for the fingertip compression; however, this may not be consistent with other clinical researchers. For example, Hernandez et al. conducted a large scale randomized controlled clinical trial [1] and they used 10 s for the fingertip compression. There have been debates regarding a standardized method of CRT measurements. Kawaguchi et al. [17] reported that a compression time from 1 to 6 s did not affect the number of CRT in healthy volunteers. They used an optic color sensor to objectively measure CRT. Alsma et al. [6], in their clinical study, showed slightly shorter CRT by a compression time of 5 s compared with 15 s (2.3 [95% CI, 2.2–2.3] vs. 2.4 [2.4–2.5]). This trend might become more remarkable as patients had prolonged CRT (3.5 [3.3–3.7] vs. 3.9 [3.6–4.1]). We would like to highlight the impact of developing a clinical device that allows for objective CRT measurement and the importance of standardizing the clinical methods to decrease the variability. We used “beep sound” to control 5 s and minimized the variability between measurements. Therefore, our internally standardized method would lead to a scientifically sound conclusion. Lastly, standardized visual CRT used in our study has been recognized as a reliable measure but this is a subjective assessment of CRT. However, we consider this method appropriate for assessing peripheral perfusion status based on the following 3 reasons in terms of its validity and reliability: [1] there have been multiple previous works by other investigators that support the reliability of standardized visual CRT [1–3] [2]; we validated the reliability of our standardized visual CRT by using the other objective measurement, such as image analysis, and a strong correlation between these measures was found in our preliminary [16] [3]; our newly developed method, BRT, may wish to be validated by other objective measurements rather than compared with subjective standardized visual CRT, and therefore, we are currently working on another analysis evaluating BRT with CRT by image analysis.

## Conclusions

BRT by a pulse oximetry sensor was an objective measurement as useful as the CRT measured by the ED physician who was trained for quantitative CRT measurements at the bedside. The results of this study demonstrate that a normal pulse oximetry sensor attached to the fingertip can be an alternative to the manual CRT in ED patients.

## Data Availability

The de-identified dataset is held by the corresponding author and the sponsor, and data may be made available in part for secondary analysis by third parties; access will be considered on a case by case basis under our corporate policy.

## Abbreviations

CRT: Capillary refill time
ED: Emergency department
BRT: Blood refill time
NIRS: Near-infrared spectroscopy
Hb: Hemoglobin
Ht: Hematocrit
CBC: Complete blood count
CRP: C-reactive protein
ESR: Erythrocyte sedimentation rate
AST: Aspartate aminotransferase
LDH: Lactate dehydrogenase
Cre: Creatinine
BUN: Blood urea nitrogen
TP: Total protein
Alb: Albumin
CI: Confidence interval
AUC: Area under the curve
ROC: Receiver operator characteristic
CV: Coefficient of variation
ICC: Intra-class coefficient
